# TRPC3-Nox2 Protein Complex Formation Increases the Risk of SARS-CoV-2 Spike Protein-Induced Cardiomyocyte Dysfunction through ACE2 Upregulation

**DOI:** 10.3390/ijms24010102

**Published:** 2022-12-21

**Authors:** Yuri Kato, Kazuhiro Nishiyama, Jae Man Lee, Yuko Ibuki, Yumiko Imai, Takamasa Noda, Noriho Kamiya, Takahiro Kusakabe, Yasunari Kanda, Motohiro Nishida

**Affiliations:** 1 Graduate School of Pharmaceutical Sciences, Kyushu University, Fukuoka 812-8582, Japan; 2 Laboratory of Creative Science for Insect Industries, Faculty of Agriculture, Kyushu University, Fukuoka 819-0395, Japan; 3 Graduate Division of Nutritional and Environmental Sciences, University of Shizuoka, Shizuoka 422-8526, Japan; 4 Laboratory of Regulation for Intractable Infectious Diseases, Center for Vaccine and Adjuvant Research (CVAR), National Institutes of Biomedical Innovation Health and Nutrition (NIBIOHN), Osaka 567-0085, Japan; 5 Department of Psychiatry, National Center of Neurology and Psychiatry, Tokyo 187-8551, Japan; 6 Integrative Brain Imaging Center, National Center of Neurology and Psychiatry, Tokyo 187-8551, Japan; 7 Department of Neuropsychopharmacology, National Institute of Mental Health, National Center of Neurology and Psychiatry, Tokyo 187-8553, Japan; 8 Department of Brain Bioregulatory Science, The Jikei University Graduate School of Medicine, Tokyo 105-8461, Japan; 9 Department of Applied Chemistry, Graduate School of Engineering, Kyushu University, Fukuoka 819-0395, Japan; 10 Division of Biotechnology, Center for Future Chemistry, Kyushu University, Fukuoka 819-0395, Japan; 11 Laboratory of Insect Genome Science, Faculty of Agriculture, Kyushu University, Fukuoka 819-0395, Japan; 12 Division of Pharmacology, National Institute of Health Sciences (NIHS), Kawasaki 210-9501, Japan; 13 National Institute for Physiological Sciences, Exploratory Research Center on Life and Living Systems, National Institutes of Natural Sciences, Okazaki 444-8787, Japan

**Keywords:** SARS-CoV-2, transient receptor potential canonical, NADPH oxidase, protein–protein interaction, chemical stress

## Abstract

Myocardial damage caused by the newly emerged coronavirus (SARS-CoV-2) infection is one of the key determinants of COVID-19 severity and mortality. SARS-CoV-2 entry to host cells is initiated by binding with its receptor, angiotensin-converting enzyme (ACE) 2, and the ACE2 abundance is thought to reflect the susceptibility to infection. Here, we report that ibudilast, which we previously identified as a potent inhibitor of protein complex between transient receptor potential canonical (TRPC) 3 and NADPH oxidase (Nox) 2, attenuates the SARS-CoV-2 spike glycoprotein pseudovirus-evoked contractile and metabolic dysfunctions of neonatal rat cardiomyocytes (NRCMs). Epidemiologically reported risk factors of severe COVID-19, including cigarette sidestream smoke (CSS) and anti-cancer drug treatment, commonly upregulate ACE2 expression level, and these were suppressed by inhibiting TRPC3-Nox2 complex formation. Exposure of NRCMs to SARS-CoV-2 pseudovirus, as well as CSS and doxorubicin (Dox), induces ATP release through pannexin-1 hemi-channels, and this ATP release potentiates pseudovirus entry to NRCMs and human iPS cell-derived cardiomyocytes (hiPS-CMs). As the pseudovirus entry followed by production of reactive oxygen species was attenuated by inhibiting TRPC3-Nox2 complex in hiPS-CMs, we suggest that TRPC3-Nox2 complex formation triggered by panexin1-mediated ATP release participates in exacerbation of myocardial damage by amplifying ACE2-dependent SARS-CoV-2 entry.

## 1. Introduction

A new pandemic of pneumonia caused by severe acute respiratory syndrome coronavirus 2 (SARS-CoV-2) emerged in 2019 and rapidly spread worldwide in early 2020 [1,2]. Severe prognostic symptoms have become a global problem and the development of effective treatments and drugs is urgently needed [3]. The coronaviruses are known for their impact on the respiratory tract, but SARS-CoV-2 can invade and infect the heart, and lead to a diverse spectrum of cardiac manifestations, including inflammation (myocarditis), arrhythmias, heart attack-like symptoms, and heart failure [4,5]. The tropism of organs has been studied from autopsy specimens: SARS-CoV-2 genomic RNA was highest in the lungs, but the heart, kidney, and liver also showed substantial amounts [6]. In fact, above 1000 copies of SARS-CoV-2 virus were detected in the heart of 31% patients who died [7]. It has been reported that the expression levels of SARS-CoV-2 receptor, angiotensin-converting enzyme 2 (ACE2), are upregulated in COVID-19 patients [8]. Although many risk factors for severe COVID-19 are reported, such as smoking, anti-cancer drug treatment, hyperglycemia, aging, and pre-existing cardiovascular diseases [9,10,11], the causal relationship between these risk factors and potential heart manifestations is unclear.

The cardiovascular and lung tissues highly express ACE2 proteins and physiologically balance the status of the renin-angiotensin (Ang) system by degrading Ang II and generating Ang 1–7 [12]. SARS-CoV-2 uses its spike (S) glycoprotein to bind with ACE2, and mediate membrane fusion and virus entry [13]. Before ACE2 receptor-dependent syncytium formation (i.e., cell–cell fusion), the surface S proteins are cleaved and activated by transmembrane protease serine proteases. The ACE2-dependent SARS-CoV-2 virus entry is reportedly achieved through endocytosis regulated by phosphatidylinositol 3-phosphate 5-kinase [14], the main enzyme synthesizing phosphatidylinositol-3,5-bisphosphate (PI(3,5)P_2_) in early endosome [15], two-pore channel subtype 2, a major downstream effector of PI(3,5)P_2_, and cathepsin L (and possibly B) [16,17], a cysteine protease that cleaves S protein to facilitate virus entry in lysosome. Increased expression of these factors is thought to cause the aggravation of COVID-19 symptoms. The myocardial ACE2 expression levels are reportedly upregulated in humans with cardiovascular diseases [18,19]. Oxidative stress through NADPH oxidase (Nox) 2-mediated reactive oxygen species (ROS) production has been reported to mediate SARS-CoV-2 pseudovirus-evoked and interleukin-6-induced ACE2 upregulation in endothelial cells [19]. In addition, we reported that Nox2 protein is upregulated in pathological hearts by forming a protein complex with transient receptor potential canonical 3 (TRPC3) channel protein [20,21]. These reports suggest that TRPC3-Nox2 protein complex formation is a common target of myocardial dysfunction evoked by SARS-CoV-2 infection as well as exposure to cardiac risk factors. Therefore, we here investigate whether TRPC3-Nox2 complex formation is involved in SARS-CoV-2-induced myocardial dysfunction and ACE2 upregulation caused by cardiac risk factors. We demonstrate that targeting inhibition of TRPC3-Nox2 complex is a new strategy for the prevention of cardiac severity after COVID-19.

## 2. Results

### 2.1. SARS-CoV-2 Pseudovirus Infection Induces Myocardial Dysfunction

**Figure 1:**
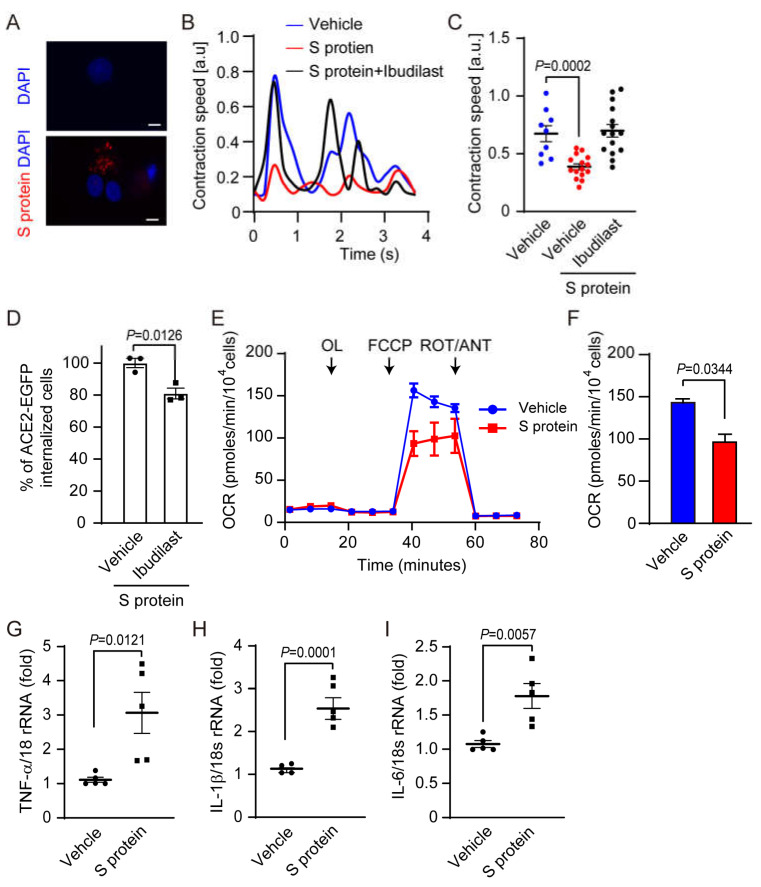
Exposure of NRCMs to SARS-CoV-2 pseudovirus causes contractile and metabolic dysfunctions and cytokine productions. (**A**) Representative images of internalization of HiLyte Fluor 555-labeled S protein (red) in NRCMs co-stained with DAPI (blue). Scale bars = 10 µm. (**B**) Spontaneous contraction speed of NRCMs by exposure to S protein (50 nM) for 3 h with (black) or without (red) ibudilast, or exposure to vehicle (blue). (**C**) Peak contraction speeds of NRCMs treated with (black) or without (red) ibudilast (100 nM) 1 h before S protein (50 nM) exposure for 3 h. Those of vehicle exposed NRCMs (control) are shown in blue. (**D**) ACE2-EGFP internalization treated with ibudilast (100 nM) 1 h before S protein for 3 h in HEK293T cells. (**E**) Oxygen consumption rate (OCR) of NRCMs stimulated with S protein (red) or PBS (blue) for 24 h. (*n* = 3 independent experiments) OL: oligomycin; FCCP: carbonyl cyanide p-[trifluoromethoxy]-phenylhydrazone; ROT: Rotenone; ANT: antimycin. (**F**) Average maximal OCR in (**E**). (**G**–**I**) cytokine mRNA expressions in NRCMs treated with S protein for 3 h. All data are shown as mean ± SEM; *n* = 3–5. Significance was imparted using *t*-test and one-way ANOVA followed by Tukey’s comparison test.

We evaluated the effect of SARS-CoV-2 on cardiomyocyte functions using S glycoprotein pseudoinfection model [22]. ACE2-EGFP-expressing HEK293T cells showed a marked ACE2 internalization by HiLyte FluorTM555-labeled S protein [23] (Figure 1A). We previously reported that the treatment of cardiomyocytes with doxorubicin (Dox), an anti-cancer drug, promotes complex formation of TRPC3 protein, a major component of receptor-activated cation channels, with Nox2 [20,21], and extracellular ATP released from neonatal rat cardiomyocytes (NRCMs) evoked by stresses such as Dox and nutrient deficiency induces formation of TRPC3-Nox2 protein complex via P2Y_2_ receptor stimulation and resultant ROS production in NRCMs [24]. Nox2-dependent ROS production contributes to pathological myocardial atrophy. We also identified that ibudilast, an anti-inflammatory drug used in the treatment of asthma and stroke, has the potency to inhibit TRPC3-Nox2 protein complex formation [25]. Exposure to S protein reduced spontaneous contraction speed of NRCMs, which was canceled by ibudilast treatment (Figure 1B,C). Internalization of ACE2 evoked by S protein exposure was suppressed by ibudilast pretreatment in ACE2-EGFP-expressing HEK293T cells. (Figure 1D). Indeed, S protein exposure also reduced mitochondrial maximal respiration of NRCMs (Figure 1E,F). Patients with severe COVID-19 have been reported to promote the inflammatory response via cytokine storm [26,27]. S protein entry increased mRNA expression levels of inflammatory cytokine (TNF-α, IL-1β, and IL-6) (Figure 1G–I). These data suggested that internalization of pseudovirus S protein can mimic the phenotype of myocardial abnormality caused by SARS-CoV-2 infection in humans.

### 2.2. TRPC3/Nox2 Complex Formation Involves in Increasing in ACE2 Expression

**Figure 2:**
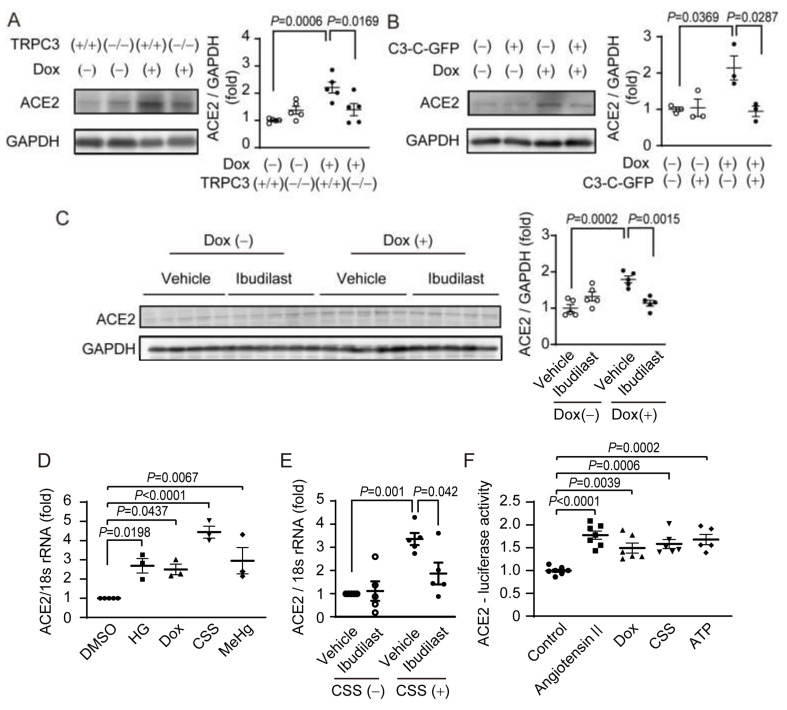
Involvement of TRPC3-Nox2 protein complex formation in pathological ACE2 upregulation in rodent hearts and cardiomyocytes. (**A**–**C**) Expression of ACE2 proteins in Dox-treated TRPC3(+/+) and TRPC3(−/−) mouse hearts (**A**), Dox-treated mouse with cardiomyocyte-specific expressing GFP-fused inhibitory peptide (C3-C) against TRPC3-Nox2 interactions (**B**), and Dox-treated mice with ibudilast (10 mg/kg/day) or vehicle (**C**). (**D**,**E**) ACE2 mRNA expressions in NRCMs exposed to several cardiac risk factors for 24 h. HG: high glucose (25 mM); Dox: doxorubicin (1 µM); CSS: cigarette sidestream smoke-containing medium (1%); and MeHg (500 nM). (**E**) Effect of ibudilast (10 µM) on CSS-exposed increase in ACE2 mRNA expression. (**F**) ACE2 transcription activities in NRCMs treated with Ang II (1 µM) and ATP (1 mM) for 24 h. All data are shown as mean ± SEM; *n* = 3–8. Significance was imparted using two-way ANOVA and one-way ANOVA followed by Tukey’s comparison test.

Next, we examined whether TRPC3-Nox2 complex formation participates in ACE2 expression of rodent hearts. Interestingly, we found that ACE2 protein abundance was markedly increased in Dox-treated mouse hearts (Figure 2A), and this ACE2 upregulation was canceled by systemic deletion of trpc3 gene or cardiomyocyte-specific overexpression of inhibitory peptide (C3-C-GFP) of TRPC3-Nox2 protein complex [21] (Figure 2A,B). These ACE2 upregulations were suppressed by co-treatment with ibudilast (Figure 2C). Additionally, ACE2 mRNA expression levels were increased in NRCMs by stresses such as hyperglycemia, Dox treatment, and exposure to cigarette sidestream smoke (CSS) and MeHg [28], an environmentally toxic heavy metal found in tuna (Figure 2D). Ibudilast inhibited the upregulation of ACE2 expression by CSS (Figure 2E). Exposure of NRCMs to Dox and CSS also increased the transcriptional activity of ACE2, like exposure to Ang II stimulation (Figure 2F). These data suggest that the formation of TRPC3-Nox2 protein complex contributes to increase in ACE2 gene expression levels caused by several stresses that are known as risk factors for cardiovascular diseases.

### 2.3. Chemical Stresses Accelerate Internalization of S Protein in Cardiomyocytes

**Figure 3:**
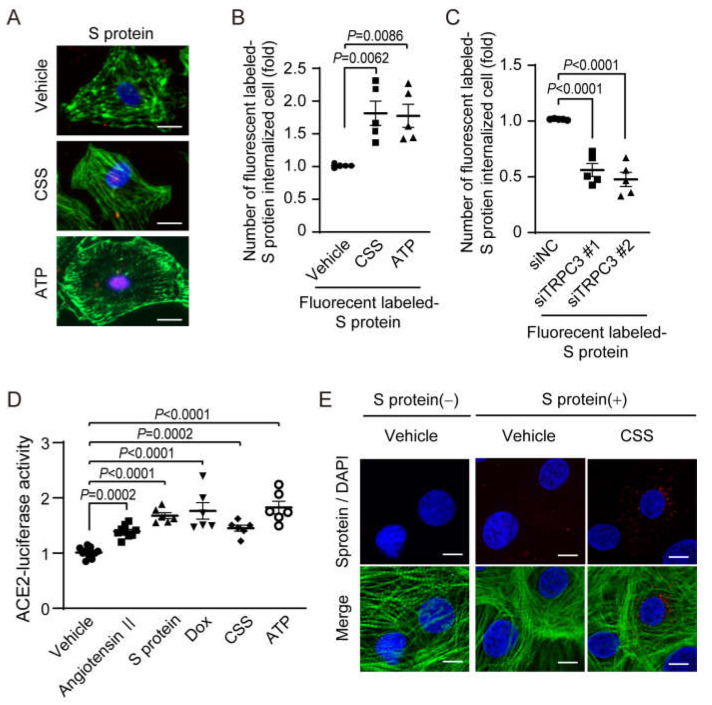
Enhanced S protein entry in NRCMs and hiPS-CMs by exposure to CSS and ATP. (**A**) Representative images of S protein incorporation in NRCMs by exposure to CSS (1%) and ATP (1 mM) for 24 h before stimulating by HiLyte Fluor 555-labeled S protein (50 nM) for 3 h (red). The S protein-exposed NRCMs were fixed and co-stained with phalloidin (green) and DAPI (blue). Scale bars = 10 µm. (**B**) Number of HiLyte FluorTM^555^-labeled-S protein-incorporated NRCMs in (**A**). (**C**) Number of S protein-incorporated NRCMs with or without silencing TRPC3. (**D**) ACE2 transcriptional activities in hiPS-CMs. hiPS-CMs were treated with Ang II (1 µM), ATP (1 µM), Dox (1 µM), and CSS (1%) for 24 h. (**E**) Representative images of S protein incorporation in hiPS-CMs exposed to CSS (1%) for 24 h before stimulating with HiLyte Fluor 555-labeled S protein (50 nM) for 3 h (red). The hiPS-CMs were co-stained with phalloidin (green) and DAPI (blue). Scale bars = 10 µm. All data are shown as mean ± SEM; *n* = 5–8. Significance was imparted using one-way ANOVA followed Tukey’s comparison test.

As chemical stresses, such as Dox and CSS exposures, strongly increased ACE2 gene expression levels, we next investigated whether these chemical stresses enhance SARS-CoV-2 pseudovirus entry to NRCMs. Treatment with fluorescently labeled S protein after pre-exposure to CSS or ATP, one of the major damage-associated molecular patterns (DAMPs) [29], significantly increased the number of S protein-incorporated cells (Figure 3A,B). The number of S protein-incorporated NRCMs were decreased by silencing TRPC3 gene (Figure 3C). The increases in ACE2 transcriptional activities by the exposure to Dox, CSS, ATP, S protein, and Ang II stimulation were also observed in hiPS-CMs (Figure 3D). Pre-exposure of hiPS-CMs to CSS also promoted the incorporation of fluorescence-labeled S proteins (Figure 3E). As smoking and anti-cancer drug treatment are also known as risk factors of COVID-19 severity, these results strongly suggest that external stresses caused by risk factors of COVID-19 enhance SARS-CoV-2 entry to human and rodent cardiomyocytes through ACE2-dependent internalization pathway.

### 2.4. S Protein Pseudovirus-Induced ATP Release via Panx1 Causes ROS Production

**Figure 4:**
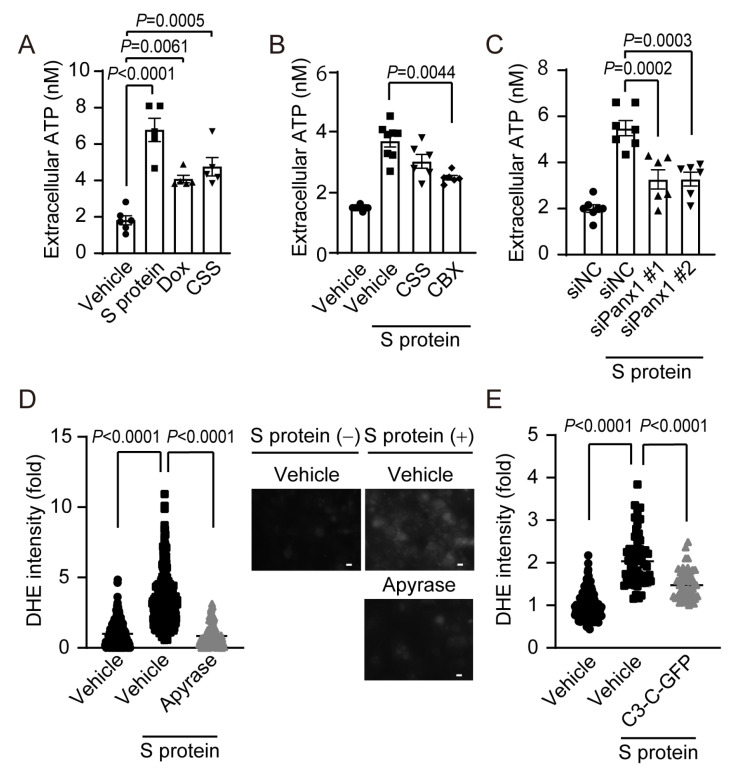
Extracellular ATP release through Panx1 mediates S protein-evoked ROS production. (**A**) ATP release evoked by the exposure of hiPS-CMs to S protein (50 nM), CSS (1%), and Dox (1µM) for 24 h. (**B**) Effects of CSS (1%) and CBX (30 µM) pretreatment for 1 h on S protein-exposed ATP release from hiPS-CMs. (**C**) Effect of Panx1 knockdown on S protein-exposed ATP release. (**D**) Effects of apyrase (2 U/mL for 1 h) on S protein-induced ROS production (white). Scale bars = 10 µm. (**E**) Effects of C3-C-GFP on S protein-induced ROS production. All data are shown as mean ± SEM; *n* = 5–8. Significance was imparted using one-way ANOVA followed Tukey’s comparison test.

As the extracellular ATP released by hypoxic and starvation stress triggers formation of TRPC3-Nox2 protein complex in NRCMs [24], we examined whether ATP release from hiPS-CMs was induced by the pseudovirus exposure and chemical stresses. Extracellular ATP concentration was significantly increased by the exposure to S protein, CSS and Dox (Figure 4A). Treatment with carbenoxolone (CBX), an inhibitor of ATP-permeable pannexin1 (Panx1), attenuated the S protein-evoked increase in extracellular ATP level (Figure 4B). Silencing Panx1 by two independent siRNAs suppressed the S protein-evoked ATP release (Figure 4C). S protein exposure also increased ROS production, which was canceled by the treatment with apyrase, an ATP-degrading enzyme (Figure 4D). In addition, overexpression of inhibitory peptide (C3-C-GFP) of TRPC3-Nox2 protein complex reduced ROS production in hiPS-CMs (Figure 4E).

### 2.5. ATP Release Evoked by S Protein Internalization Promotes TRPC3-Nox2 Complex Formation in Cardiomyocytes

**Figure 5:**
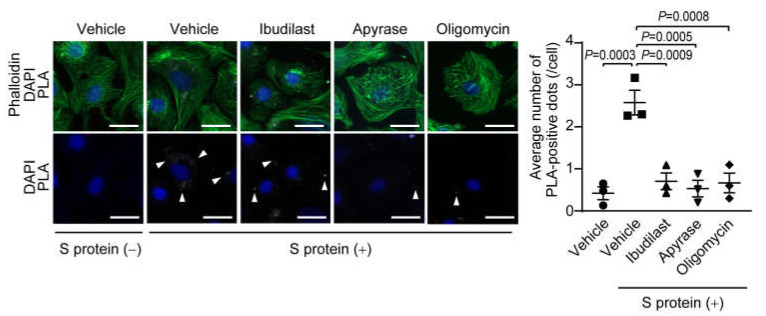
S protein exposure induces TRPC3-Nox2 complex formation through ATP release. Representative images of TRPC3-Nox2 complex formation in NRCMs treated 3 h after S protein (50 nM) exposure (left). NRCMs were treated with Apyrase (2 U/mL) or oligomycin (1.5 µM) 1 h before S protein exposure. PLA signals between TRPC3 and Nox2 antibodies are shown as white spots (arrowheads), counterstained with phalloidin (green) and DAPI (blue). Scale bars = 20 µm. Average number of PLA signals for each cell was quantified in left panel (right). At least 50 cells were counted. All data are shown as mean ± SEM; *n* = 3. Significance was imparted using one-way ANOVA followed Tukey’s comparison test.

Finally, we confirmed using proximity ligation assay (PLA) that the formation of TRPC3-Nox2 protein complex was promoted by S protein exposure in NRCMs (Figure 5). S protein-evoked TRPC3-Nox2 complex formation was suppressed by ibudilast. Treatment with apyrase and oligomycin, an ATP synthase inhibitor, reduced the number of PLA-positive dots evoked by S protein exposure (Figure 5). These results strongly suggest that pseudovirus-evoked ATP release via Panexin1 hemi-channels contributes to enhancement of SARS-CoV-2 entry and ROS production in human cardiomyocytes through formation of TRPC3-Nox2 protein complex.

## 3. Discussion

Severe COVID-19 cases are reported to cause not only lung damage, and gastrointestinal symptoms but also myocardial dysfunction such as myocardial injury, myocarditis, arrhythmias, and heart failure [30,31,32,33]. Production of cytokines, ROS, and DAMPs including ATP have been thought to participate in these symptoms [29,32,34,35,36]. In addition, epidemiological studies have clarified that smoking, obesity, and medical history, such as cancer and anti-cancer drug treatment, diabetes and hypertension, are major risk factors of severe COVID-19 [10,37]. However, it is unclear how these risk factors contribute to the severe symptoms including myocardial dysfunction. In this study, we demonstrated that the increase in ACE2 gene expression levels by the exposure to external stresses were well-associated with enhancement of SARS-CoV-2 pseudovirus entry followed by ROS and cytokine mRNA productions, metabolic and contractile dysfunctions in human and rat cardiomyocytes.

It was previously reported that beating of hiPS-CMs was significantly suppressed after SARS-CoV-2 infection [38,39]. We established SARS-CoV-2 pseudovirus infection model using NRCMs and revealed that S protein exposure can mimic the SARS-CoV-2-induced contractile dysfunction of cardiomyocytes (Figure 1). In addition to contractile dysfunction, mitochondria are attracted attention as an intracellular virus sensor and mitochondrial damage is associated with cellular dysfunctions of energy metabolism, intracellular Ca^2+^ handling, and redox homeostasis [40,41]. Indeed, SARS-CoV-2 infection is thought to promote mitochondrial dysfunction [39,42,43]. We found that mitochondrial maximal respiration was reduced, and mRNA expression levels of inflammatory cytokines were increased in NRCMs after S protein exposure (Figure 1). These results suggest that S protein exposure in NRCMs also mimics the phenotypes of metabolic dysfunction and inflammation in human hearts caused by SARS-CoV-2 infection.

ACE2 gene expression levels are reportedly elevated in the organs of SARS-CoV-2-infected patients and the result of GWAS analysis indicates that people with low ACE2 gene expression level tended to be less sensitive to the SARS-CoV-2 virus [8,44]. Thus, the expression level of ACE2 protein must be one of the key determinants for COVID-19 aggravation. We found that exposure to several environmental stresses considered as causes of cardiac risk factors upregulates ACE2 and promotes SARS-CoV-2 pseudovirus entry to cardiomyocytes (Figure 2 and Figure 3). We also suggest that the extracellular ATP-mediated formation of TRPC3-Nox2 protein complex contributes to ACE2 upregulation. TRPC3 protein is a component of receptor-operated/diacylglycerol-activated cation-permeable channels present on the cell membrane and is ubiquitously expressed [45,46]. Nox2 protein is also distributed broadly in many tissues and organs [47]. We reported that the physical interaction between TRPC3 and Nox2 proteins leads to the increased Nox2 protein-dependent ROS production and pathological cardiac remodeling, such as myocardial atrophy and interstitial fibrosis [21]. Pharmacological perturbation of TRPC3-Nox2 protein complex by ibudilast and inhibitory peptide, C3-C-GFP, prevented the increase in ACE2 expression and S protein incorporation by exposure to chemical stresses. As S protein exposure also induces TRPC3-Nox2 complex formation and increases ACE2 expression, SARS-CoV-2 may amplify its incorporation to cardiomyocytes through ACE2 upregulation. ACE2 and cytokine gene expressions are positively regulated by Apelin, SIRT1, and Foxo [48,49]. As the protein abundances of these transcription factors are reported to be increased by diabetes, smoking, and cancer [48,50,51], gene transcription-dependent amplification of SARS-CoV-2 virus entry may be important to cause COVID-19 severity in patients with risk factor(s).

**Figure 6:**
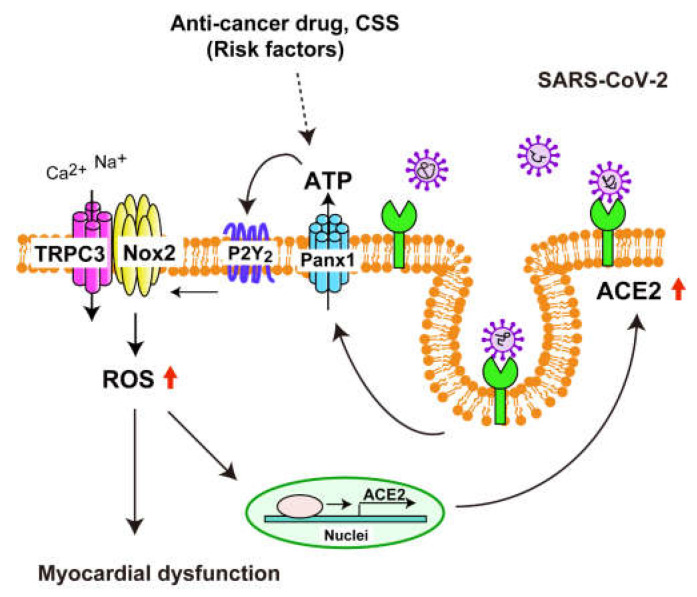
Hypothetical model of ACE2 upregulation in cardiomyocytes by the exposure to SARS-CoV-2 and cardiac risk factors in cardiomyocytes. Exposure to SARS-CoV-2, smoking, and anti-cancer drug treatment commonly upregulates ACE2 protein expression by ATP release through Panx1. The released ATP then induces TRPC3-Nox2 complex formation and promotes ROS production probably through P2Y_2_ receptor stimulation [24]. The formation of TRPC3-Nox2 protein complex may lead to SARS-CoV-2-induced cardiac severity by amplifying ROS-dependent signaling in cardiomyocytes.

Recently, SARS-CoV-2 infection was reported to release ATP through Panx1 in lung cells [52]. In human immunodeficiency virus (HIV) infection, Panx1 has been reported to promote intracellular uptake of the HIV virus via ATP release [53]. We found that Panx1 channel-dependent ATP release is caused after exposure of cardiomyocytes to SARS-CoV-2 pseudovirus, and that the released extracellular ATP contributes to TRPC3-Nox2 protein complex formation and ROS production of cardiomyocytes (Figure 4 and Figure 5). In addition to S protein exposure, exposure to chemical stresses considered as risk factors for severe COVID-19 also induces ATP release, and the released ATP increases ACE2 expression through TRPC3-Nox2 complex formation (Figure 6). Not only pseudovirus S protein exposure but also inflammatory or hypoxic stress reportedly increase the ACE2 expression level through alternative signaling pathways including hypoxia inducible factor 1a (HIF1a) [54,55], but we have not investigated whether Panx1-mediated ATP release and/or TRPC3-Nox2 axis participates in these alternative pathways. Further studies will be necessary to determine whether Panx1-mediated ATP release and TRPC3-Nox2 complex formation are essential for the increase in ACE2 expression. Furthermore, increased ACE2 expression due to various stimuli is thought to increase the risk of SARS-CoV-2 infection in cells, while increasing anti-inflammatory effect mediated by the intrinsic enzymatic action of ACE2, converting pro-inflammatory Ang II into anti-inflammatory Ang 1–7 [49]. Therefore, it is necessary to consider a drug discovery strategy to inhibit ACE2-mediated SARS-CoV-2 entry without inhibiting ACE2 enzymatic activity as well as endogenous antiviral immunity.

Myocarditis and pericarditis have been reported after COVID-19 mRNA vaccination [56,57]. To stimulate adaptive immune response, the COVID-19 vaccine generates S protein within host cells. As the pseudovirus S protein is sufficient to cause cardiomyocyte dysfunctions, it would be possible to consider the involvement of TRPC3-Nox2 protein complex in the incidence of adverse effect by mRNA vaccination. As the USA has initiated a clinical trial of ibudilast to treat acute respiratory distress syndrome (ARDS) in COVID-19 patients [58], further studies focusing on TRPC3-Nox2 protein–protein interactions may establish a new strategy for the prevention or treatment against human COVID-19 severity and vaccine side effects.

## 4. Materials and Methods

### 4.1. Cell Culture

ACE2-EGFP-expressing HEK293T cells were cultured in Dulbecco’s modified eagle medium (DMEM) supplemented with 10% FBS and 1% penicillin and streptomycin [23]. Human-derived iPS cardiomyocytes (hiPS-CMs) products, iCell Cardiomyocytes2, were purchased from FUJIFILM Cellular Dynamics, Inc. (Osaka, Japan) and maintained according to the manufacturer’s instructions. Isolation of neonatal rat cardiomyocytes (NRCMs) were performed as described previously [20]. Plasmid DNAs were transfected into HEK293 cells, hiPS-CMs and NRCMs with lipofectamine 3000 (Thermo Fisher Scientific, Waltham, MA, USA) or ViaFect Transfection Reagent (Promega, Madison, WI, USA) according to manufacturer’s instructions.

### 4.2. S Protein and HiLyte Fluor 555-Labeled S Protein

Recombinant SARS-CoV-2 spike protein (S protein) were purified using the baculovirus-silkworm system [22]. Purified S protein was chemically labeled with a fluorescent probe using HiLyte FluorTM 555 Labeling Kit-NH2 (Dojindo, Kumamoto, Japan).

### 4.3. ACE2-Internalization Assay with S Protein [59]

ACE2-EGFP-expressing HEK293T cells (1.5 × 10^4^ cells/well) were incubated at least 24 h before addition of S protein. In each well, the cells were incubated with S protein (50 nM) or HiLyte Fluor 555-labeled S protein (100 nM) for 3 h at 37 °C, 5% CO2. Cells were fixed in 4% paraformaldehyde for 10 min to stop the reaction and mounted with ProLong Diamond Antifade Mountant containing DAPI (Thermo Fisher Scientific, Waltham, MA, USA). Imaging was performed on a BZ-X800 microscope (Keyence, Osaka, Japan). ACE2-internalized cells were counted in each section and normalized without S protein as a control.

### 4.4. Observation of Spontaneous Contractility

NRCMs (4 × 10^5^ cells/well) were seeded on Φ 3.5 cm glass bottom dish. The cells were incubated with S protein (50 nM) for 3 h at 37 °C, 5% CO2. Microscopy images of NRCMs were recorded for 13 s under pacing conditions. Electrical stimulation was applied for 10 msec every 1 sec at 4 V (SEN-3301, NIHON KOHDEN, Tokyo, Japan).

### 4.5. The Analysis of Contractility

Imaging was performed at 6 frames per second on a BZ-X800 microscope (Keyence). Each video was analyzed by Image J software as previously described [60].

### 4.6. Mitochondrial Bioenergetics Analysis

Oxygen consumption rate (OCR) were assessed using a XFp Extracellular Flux Analyzer (Seahorse Bioscience, North Billerica, MA, USA). The hiPS-CMs were seeded onto the plates with a density of 1.0 × 10^4^ cells/well. The cells were treated with or without clomipramine (100 nM) for 1 h followed by S protein (50 nM) for 24 h. Prior to analysis, cells were incubated for 1 h in XF Base Medium supplemented with 25 mM d-glucose, 1 mM pyruvate and 2 mM glutamine. After measurements of baseline OCR, measurements were made following sequential automatic injections of a final concentration of 10 µM oligomycin, 2 µM carbonyl cyanide p-[trifluoromethoxy]-phenylhydrazone (FCCP), 10 µM rotenone, and 10 µM antimycin A. After measurements of OCR, cells were fixed in 4% paraformaldehyde and washed twice with PBS, permeabilized using 0.1% Triton X-100 in PBS for 5 min, and treated with 3% BSA in PBS for 1 h at room temperature. Then, nuclei were stained with DAPI for 1 h, cell images were captured, and the number of cells was counted using BZ-X800 microscope (Keyence). All values for OCR were normalized to number of cells present in each well.

### 4.7. Isolation of mRNA and Real-Time PCR

NRCM (3.0 × 10^4^ cells/well) were incubated at least 24 h before treatment. Total RNA was isolated with a TRI reagent (Sigma, Burligton, MA, USA) from NRCM treated by S protein for 3 h. cDNA was synthesized with a ReverTra Ace qPCR RT Master Mix kit (Toyobo, Osaka, Japan). Quantitative real-time PCR was performed with ABI prism 7500 Real-Time PCR system (Thermo Fisher Scientific, Waltham, MA, USA) and KAPA SYBR FAST qPCR kit (Roche, Basel, Switzerland). TNF-α primers were forward 5′- AAATGGGCTCCCTCTCATCAGTTC-3′ and reverse 5′- TCTGCTTGGTGGTTTGCTACGAC-3′ [61]. IL-1β primers were forward 5′-CACCTCTCAAGCAGAGCACAG-3′ and reverse 5′-GGGTTCCATGGTGAAGTCAA-3′ [61]. IL-6 primers were forward 5′-TCCTACCCCAACTTCCAATGCTC-3′ and reverse 5′-TTGGATGGTCTTGGTCCTTAGCC-3′ [61]. ACE2 primers were forward 5′- GCAGATGGCTACAACTATAACCG-3′ and reverse 5′-C CCTCCTCACATAGGCATGAAGA-3′. A total of 18 s rRNA amplification were forward 5′-ATTAATCAAGAACGAAAGTCGCAGGT-3′ and reverse 5′-TTTAAGTTTCAGCTTTGCAACCATACT-3′. Data were normalized with 18 s rRNA.

### 4.8. Animals

All animal studies were conducted according to the guidelines concerning the care and handling of experimental animals, and approved by the ethic committees at National Institutes of Natural Sciences (21A057, 29 March 2021) or the Animal Care and Use Committee, Kyushu University (A21-155-0, 12 March 2021). Male C57BL/6 mice (19–23 g, 8–10 weeks old) were purchased from CLEA Japan, Inc. (Tokyo Japan). Male and female 1–3 days old Sprague–Dawley rat pups for the isolation of NRCMs were purchased from Japan SLC Inc. (Shizuoka, Japan). All mice were maintained under controlled environment (12 h light/12 h dark cycle, room temperature 21–23 °C and humidity 50–60%). The 129Sv mice with homozygous deletion of the gene encoding TRPC3 were provided by the National Institute of Environmental Health Sciences.

### 4.9. AAV-Mediated Expression of TRPC3 C-Terminal Fragment in Mouse Hearts

Preparation of Mice expressing a TRPC3 C-terminal fragment in their hearts was carried out using the previously described method [21]. The Nox2-interacting fragment from the TRPC3 C-terminus (C3-C fragment) was PCR amplified and cloned into pEGFP-N1 vector. The EGFP-fused C3-C fragment (C3-C-GFP) cassette under the control of cardiac troponin T promoter was subcloned into pZac2.1. Viral vectors encoding EGFP or C3-C-GFP were generated as described previously. The AAV vectors (1 × 1010 genomic copies) were injected into male 6-day-old C57BL/6J mice. Expression of C3-C-GFP in the LV myocardium was verified using a fluorescence microscope (BZ-X710, Keyence, Osaka, Japan) [21].

### 4.10. Animal Model

Osmotic pumps (ALZET) for sustained administration of ibudilast (10 mg/kg/day) or vehicle were implanted intraperitoneally 3 days before Dox administration. Male C57BL/6 mice were administered Dox (15 mg/kg, i.p.) or vehicle and sacrificed 2 weeks later. Hearts were removed from mice. The dosage for Dox and ibudilast was determined in reference to a previous study [21].

### 4.11. Western Blotting Assay

Hearts were homogenized in RIPA buffer containing 0.1% SDS, 0.5% sodium deoxycholate, 1% NP-40, 150 mM NaCl, 50 mM Tris-HCl (pH 7.4), and protease inhibitor cocktail (Nacalai, Kyoto, Japan). Samples (10 µg) were then fractionated by SDS-PAGE and transferred onto PVDF membranes (Millipore, Burligton, MA, USA). The membranes were blocked with Tris-buffered saline plus 0.05% Tween-20 (TBST) containing 1% BSA and incubated with primary antibodies overnight at 4 °C. Primary antibodies against ACE2 (R&D Systems), and GAPDH (FUJIFILM) were diluted with TBST containing 1% BSA. After this incubation, the membranes were washed with TBST and incubated with HRP-conjugated secondary antibodies. The blots were visualized using Western Lightning Plus ECL (PerkinElmer, Waltham, MA, USA). Blots were normalized to those obtained with antibodies against GAPDH. Images were captured with an ImageQuant LAS 4000 (GE healthcare Life Science).

### 4.12. Preparation of Cigarette Sidestream Smoke-Containing (CSS)

Preparation of CSS was carried out using the previously described method [62]. Briefly, CSS generated by the spontaneous combustion of five cigarettes (tar: 14 mg, nicotine: 1.2 mg, Japan Tobacco Co., Tokyo, Japan) was trapped in 100 mL of DMEM (100% CSS) by bubbling using a dry vacuum pump (pumping speed: 45 l/min; DA-30D; ULVAC, Kanagawa, Japan).

### 4.13. Dual Luciferase Assay

The Dual luciferase assay was performed with the Dual-Glo luciferase assay system (Promega), according to the manufacturer’s instructions. ACE2 (-1119)-luciferase plasmids (Addgene #31110)46 and pEF-Renilla luciferase plasmids were co-transfected into hiPS-CMs and NRCMs (1.7 × 10^4^ cells/well). A total of 48 h after transfection, each compound was added. The activity of both firefly and Renilla luciferases was determined 24 h after stimulation. Luciferase activity was measured using a NIVO plate reader (PerkinElmer). The luciferase activities were normalized to the Renilla luciferase activity of the internal control.

### 4.14. Gene Knock Down in NRCMs

NRCMs (2 × 10^4^ cells/well) were seeded on matrigel-coated 96 well plates. The cells were incubated with S protein (50 nM) for 3 h at 37 °C, 5% CO2. For protein knockdown, cells were transfected with siRNAs (100 nM) using Lipofectamine RNAi Max transfection reagent (Thermo Fisher Scientific) for 72 h. Rat siTRPC3#1 and siTRPC3#2 is RSS329520 and RSS329521 (Thermo Fisher Scientific). Human siPanx1#1 and siPanx1#2 is HSS119236 and HSS119237 (Thermo Fisher Scientific).

### 4.15. Extracellular ATP Assay

After incubating in serum-free DMEM with S protein (50 nM) for 3 h at 37 °C, extracellular ATP concentration in the medium were measured using ATP bioluminescent Assay kit ( Sigma, Burligton, MA, USA) according to a manufacturer’s instruction. Each compound was added 1 h before incubation with S protein.

### 4.16. Measurements of ROS Production in hiPS-CMs

Production of ROS in hiPS-CMs were measured using DHE staining. DHE staining was performed by the incubation with DHE (2 µM) for 30 min at 37 °C, 5% CO2. Cells (1.7 × 104 cells/well) were fixed in 4% paraformaldehyde and mounted with ProLong Diamond Antifade Mountant containing DAPI. Imaging was performed on a BZ-X800 microscope. The DHE florescence intensity was analyzed from at least 40 cells in each experiment using ImageJ software.

### 4.17. Plasmid DNA and Transfection

Detailed information of fragment of TRPC3 C-terminus (C3-C-GFP) was described in the previous study [20]. Plasmid DNAs were transfected to hiPS-CMs by Lipofectamine 3000 (Thermo Fisher Scientific, Waltham, MA, USA).

### 4.18. Proximity Ligation Assay (PLA) Assay

To determine TRPC3-Nox2 interaction in NRCMs, proximity ligation assay was conducted using Duolink PLA Fluorescence (Sigma Aldrich) according to the manufacturer’s instruction. After fixing and blocking, NRCMs were incubated with rabbit anti-TRPC3 (Alomone lab, Jerusalem, Israel) and mouse anti-Nox2 (Santa Cruz, Dallas, Texas, USA) followed by PLA probes incubation for 1 h. The ligation (1 h) and amplification (3 h) steps were performed in 37 °C chamber and NRCMs were nuclear stained with DAPI and phalloidin. Images were captured using a fluorescence microscope (BZ-X800, Keyence).

### 4.19. Materials

Ibudilast was purchased from Tokyo Chemical Industry (Tokyo, Japan). CBX and apyrase were purchased from Sigma Aldrich. Oligomycin was purchased from Fujifilm. Ang II purchased from Peptide Institute, INC (Osaka, Japan).

### 4.20. Statistics

G*Power3.1.9.2 software was used to calculate the sample size for each group. All results are presented as the mean ± SEM from at least 3 independent experiments. Statistical significance was determined by unpaired *t*-test for two group comparisons by one-way analysis of variance (ANOVA) with Tukey’s test for comparison, by two-way ANOVA followed with Tukey’s comparison test for comparison among three or more groups. Statistical analysis was performed using GraphPad Prism 9.0 (GraphPad Software, LaJolla, CA, USA). Some results were normalized to control to avoid unwanted sources of variation.

## Data Availability

Not applicable.
